# A Conversation with Shankar Balasubramanian

**DOI:** 10.1021/acscentsci.2c00857

**Published:** 2022-07-29

**Authors:** Rachel Brazil

As one of the inventors of next-generation DNA sequencing, Sir Shankar Balasubramanian could claim to be responsible for a revolution in the life sciences.

**Figure:**
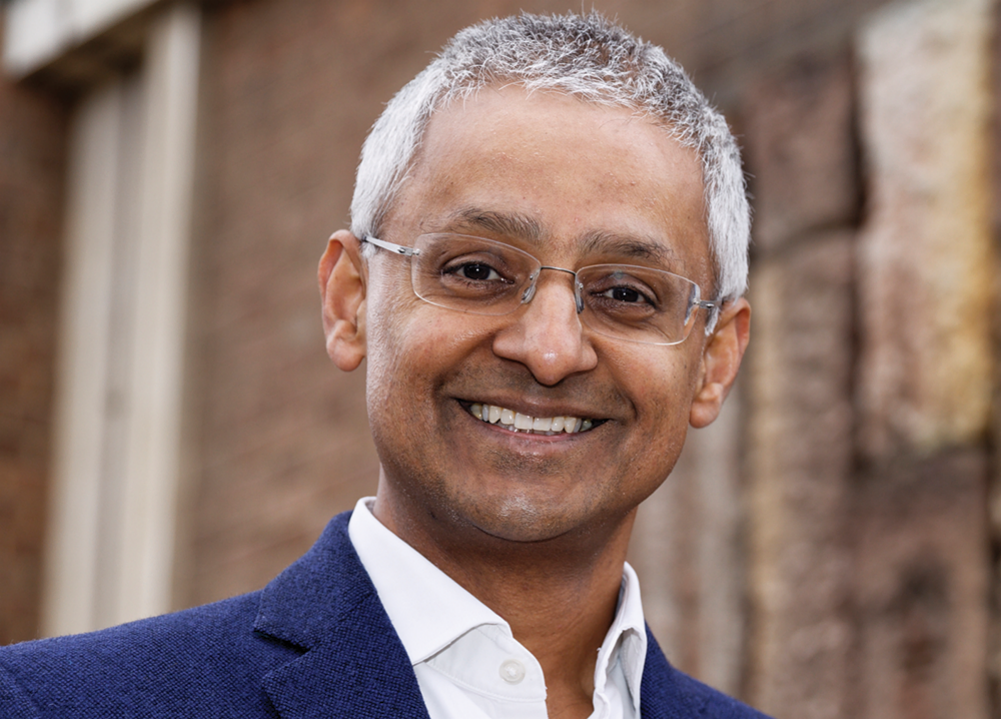
Credit: Nathan Pitt/University of Cambridge

Balasubramanian and chemist Sir David Klenerman, both at the University of Cambridge, founded the start-up Solexa in 1998, and in 2006 released their first commercial genome analyzer, able to sequence billions of DNA bases in parallel by detecting color-coded nucleotides as they are added to growing DNA strands. Solexa was acquired by Illumina, which as of last year had an 80% share of the global DNA sequencing market. In 2012, Balasubramanian launched Cambridge Epigenetix, a company developing technologies that sense DNA methylation and other modifications of DNA’s four nucleotide bases during sequencing.

He has stayed in academia and continued working on many of the fundamental questions related to the structure of DNA molecules. One of his main areas of study is G-quadruplexes, which consist of four guanine bases often on a single DNA strand that come together and cause DNA to loop back on itself twice. His group has developed methods to detect the hundreds of thousands of G-quadruplexes in human cells and are now investigating their role in regulating transcription and shaping cell programming.

Rachel Brazil spoke to Balasubramanian about his early work on next-generation sequencing and his continued fundamental work on DNA structure and function. This interview was edited for length and clarity.

## What were the biggest challenges you faced in developing your sequencing method, which ultimately led to Illumina’s sequencing system?

If you ask each type of scientist, they’ll focus on their particular area and point out the challenges. For me, the part I was most involved with was the polymerase biochemistry and the nucleotide chemistry.

But I would say perhaps the greatest overall challenge was bringing all the components together into an integrated system: going from the basic science to building a commercial system that has the accuracy and the cost characteristics to really democratize sequencing. We wanted to put this in the hands of people who could do interesting things with it.

## What other tools have you been developing to study genetic information?

Information is encrypted in DNA in many different ways. The sequence of the four genetic bases [commonly represented by the letters C, G, A, and T] is one aspect, but it’s not the only one.

Another key area, of course, is natural epigenetic modifications of DNA, such as methylation. The old way of identifying methylation sites uses the chemical reagent bisulfite. It skips over the methylated cytosines but converts unmethylated cytosines to uracils—which are actually read as thymines. That’s not great, because from a sequencing perspective, it converts your genetic alphabet to three letters rather than four. You lose genetic information to gain the epigenetic information.

What’s needed is a sequencing method that directly reads more than four letters of the DNA alphabet. You have to come up with a clever system of converting the identity of bases in a way that depends on whether they’re modified or not. There are technologies coming out, including from my company, Cambridge Epigenetix. We have methods for sequencing five letters simultaneously, and also six-letter sequencing will be coming out soon.

## Do you think genetic research will move beyond simple base pairs and focus on larger DNA structures, like G-quadruplexes?

I think we should keep an open mind to what some people call alternative DNA structures, and the G-quadruplex story is an example. We actually started the G-quadruplex work around the same time as Solexa sequencing was up and running, but it has taken longer to see more of the picture.

An important aspect that is different now is that we’ve created the tools to look into such possibilities in DNA, in chromatin, in cells. A small lab can do what the whole world couldn’t do 20 years ago.

## What are you discovering about the role of G-quadruplexes in controlling cell gene expression?

Something we wanted to understand was where G-quadruplexes form in cells. The ones that we could actually detect in chromatin were heavily enriched in regulatory regions, in particular the region upstream of transcription start sites. This fit our hypothesis that these structures are associated with transcription somehow.

The most recent work we’ve done is at the single-cell level. We think of cells as having different identities which they take on as they mature from stem cells. We’ve been able to infer the identity of a cell only by profiling its G-quadruplexes, which fits my view that G-quadruplexes are a marker of cellular identity. Our recent study has shown that as human stem cells differentiate into two different lineages, we are able to see the changes in quadruplexes and chromatin in key genes.

The next chapter is really about trying to establish details of the quadruplexes’ functions and map out pathways and protein interactions. And this still leaves open the holy grail question as to exactly what controls the formation of quadruplexes. We don’t know that yet; that’s a work in progress.

## You’re also interested in how DNA structure relates to cancer. Could understanding G-quadruplexes provide routes to new cancer therapeutics?

I think of cancer cells as being different states of healthy cells after a reprogramming of their functions. In 2014, we found that in liver and stomach cancers the cancer tissue had a much higher density of quadruplexes than a noncancerous tissue. This suggested that there was a link between G-quadruplexes and the sort of functional state of the cell.

Perturbing the epigenome is an effective strategy being deployed to treat cancers, and it’s usually through targeting the epigenetic machinery—histone deacetylases or DNA methyltransferases. I think G-quadruplexes may provide another mechanism. Many approved drugs work by interacting with DNA somewhat indiscriminately, but targeting quadruplexes is different; you’re targeting a defined feature that is linked to cellular function..

There’s more work to be done here to understand the details of the associated biological mechanism. Twenty years ago, I wasn’t sure that these structures even existed in living systems, so things have come a long way.

*Rachel Brazil is a freelance contributor to**Chemical & Engineering News**, an independent news outlet of the American Chemical Society.*

